# 530. Bamlanivimab (BAM) for SARS-CoV-2 Infection: Rates and Risk Factors for Hospitalization after Monoclonal Antibody Administration in a High-Risk Population

**DOI:** 10.1093/ofid/ofab466.729

**Published:** 2021-12-04

**Authors:** John M Curtin, Varea H Costello, Benjamin L Custer, Jason M Blaylock, Catherine F Decker, Roseanne Ressner, Sara Robinson, Wesley R Campbell, Dana M Blyth, Dana M Blyth, Anuradha Ganesan

**Affiliations:** 1 Walter Reed National Military Medical Center, North Bethesda, Maryland; 2 Walter Reed National Military Medical Center, Bethesda, Bethesda, Maryland; 3 WRNMMC/USU, Bethesda, Maryland; 4 Walter Reed National Military Medical Center, Bethesda, MD, Bethesda, Maryland; 5 Infectious Disease Clinical Research Program and the Henry M. Jackson Foundation for the Advancement of Military Medicine and Walter Reed National Military Medical Center, Bethesda, MD

## Abstract

**Background:**

In response to the ongoing COVID-19 pandemic, an emergency use authorization (EUA) was issued for neutralizing antibody therapies including BAM. Licensing trials suggest that use of BAM reduces hospitalizations when compared with placebo (1.6% vs 6.3%). However, the real world impact of BAM is not well-described. In this study, risk factors, outcomes, and hospitalization rates among high-risk outpatients presenting with mild-to-moderate COVID-19 who received BAM were examined.

**Methods:**

This is a single center retrospective analysis of all patients who received BAM monotherapy between 11/11/2020 and 3/16/2021. Electronic health records were reviewed for baseline demographics, EUA indications, comorbidities, and outcomes to include infusion reactions, hospitalizations, and deaths occurring within 29 days of BAM administration. Moderate COVID-19 was defined as having any infiltrate on chest imaging prior to BAM administration. Chi-squared or Fisher’s exact tests were used to compare categorical values as appropriate, and Mann-Whitney U for continuous variables.

**Results:**

Of the 101 patients who received BAM (median age 64 years; 21% black; 4% Hispanic; 55% male), 13 were subsequently admitted. 22 patients (22%) had moderately severe disease as evidenced by abnormal imaging. Severity on presentation, number of indications for therapy, hypertension, stroke, diabetes, and number of co-morbidities were significantly associated with subsequent admission (table 1). No patients had adverse infusion reactions. Of those hospitalized, 8 (61.5%) were for COVID-19, the median duration of hospitalization was 2 days, and 4 received guideline-directed treatment for COVID-19 (table 2).

Table 1. Factors Associated with Hospitalization Following Bamlanivimab (BAM) Administration

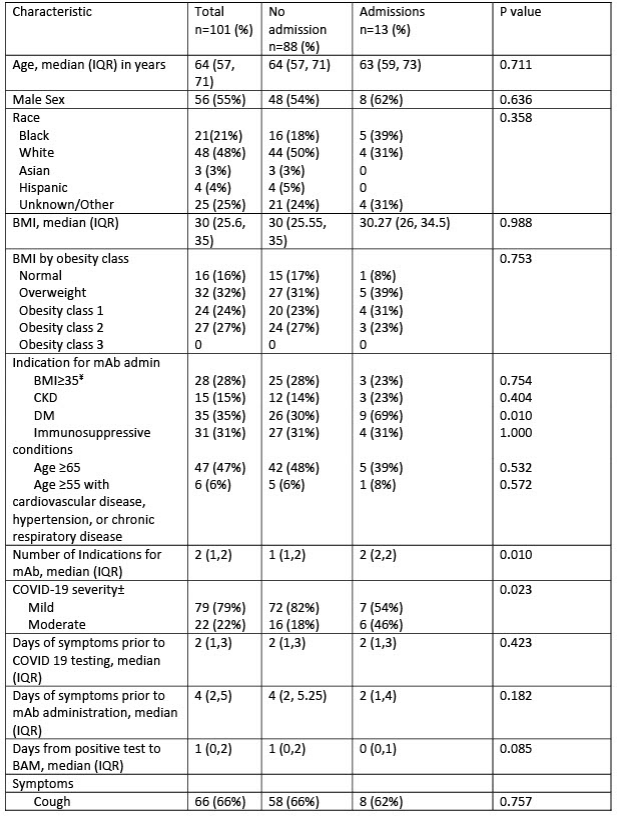

Table 1. (Continued) Factors Associated with Hospitalization Following Bamlanivimab (BAM) Administration

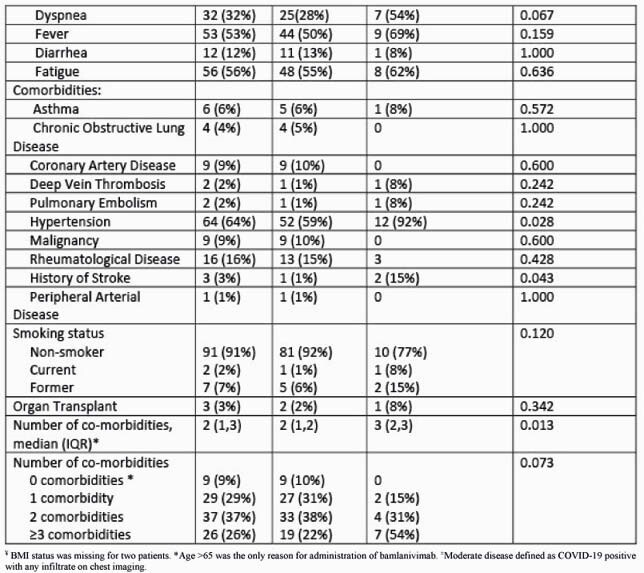

Table 2: Characteristics and Resource Utilization of Patients Hospitalized After Bamlanivimab Therapy (n=13)

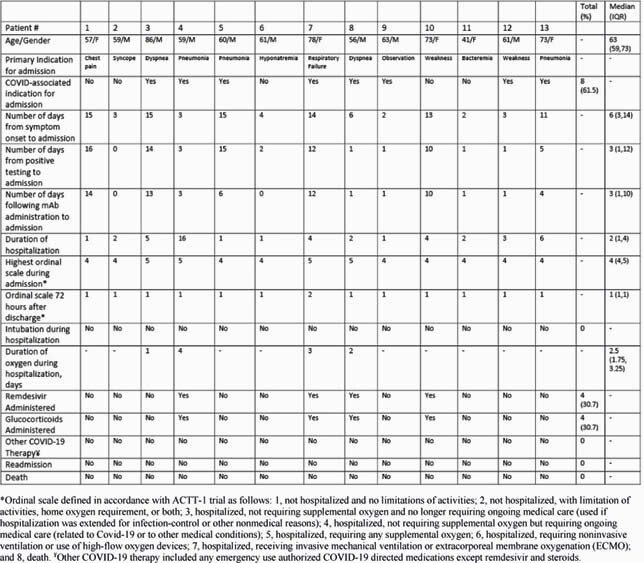

**Conclusion:**

In a high-risk population, hospitalization rates were higher than those observed in clinical trials, with 8% of subjects being admitted for COVID-19. Disease severity on presentation, multiple indications for therapy, and the presence of multiple co-morbidities were all associated with subsequent admission. Reassuringly, BAM was well tolerated, and in those requiring admission, hospitalizations were short, resource utilization was low, and there were no deaths.

**Disclosures:**

**Benjamin L. Custer, M.D.**, Alexion Pharmaceuticals (Shareholder)Armata Pharmaceuticals (Shareholder)Biomarin Pharmaceutical (Shareholder)Crispr Therapeutics (Shareholder)CVS Health Corp (Shareholder)Editas Medicine (Shareholder)Gilead (Shareholder)Glaxo Smith Kline (Shareholder)Hologic Inc (Shareholder)Merck (Shareholder)Mesoblast LTD (Shareholder)Pfizer (Shareholder)Sanofi (Shareholder)Unitedhealth Group (Shareholder)Vertex Pharmaceuticals (Shareholder) **Dana M. Blyth, MD**, Nothing to disclose

